# A Recent Lineage Split in *Cladium mariscus* Preceding the Disjunction of Two Biodiversity Hotspots: Evidence From Plastid Genomes

**DOI:** 10.1002/ece3.72456

**Published:** 2025-12-26

**Authors:** Xin‐Hui Qian, Liang‐Liang Yue

**Affiliations:** ^1^ National Wetland Ecosystem Fixed Research Station of Yunnan Dianchi Southwest Forestry University Kunming China; ^2^ National Plateau Wetlands Research Center Southwest Forestry University Kunming China; ^3^ Yunnan Key Laboratory of Plateau Wetland Conservation, Restoration and Ecological Services Southwest Forestry University Kunming China; ^4^ Dianchi Lake Ecosystem Observation and Research Station of Yunnan Province Southwest Forestry University Kunming China

**Keywords:** chloroplast complete genome, *Cladium mariscus*, Cyperaceae, phylogenetic, relaxed molecular clock

## Abstract

We report the complete chloroplast genomes of two geographically disjunct populations of *Cladium mariscus* from the Yaluzangbu Valley (Bomi) and the Hengduan Mountains (Ninglang). Phylogenomic analysis supports the basal position of *Cladium* within Cyperaceae. We identify a Late Pleistocene lineage split (~68 kya) coinciding with the MIS4 glaciation, likely facilitated by earlier northward expansion during a warm period followed by glacial isolation. The chloroplast genomes show a contracted IR region and expanded LSC region, and exhibit strong A/T bias in SSRs, indicating compositional constraints. Additionally, *C. mariscus* displays distinct genomic features, including structural variation, compared to other Cyperaceae. This study highlights the role of Quaternary climate changes in driving divergence in high‐altitude wetlands and provides valuable genomic resources for further research.

## Graphical abstract

Our complete plastome analysis of *Cladium mariscus* reveals a Late Pleistocene divergence (~68.5 kya) between disjunct Tibetan (Bomi) and Yunnanese (Ninglang) populations—predating the tectonic disjunction of the Hengduan and Yarlung Tsangpo biodiversity hotspots. Climate‐driven habitat fragmentation during MIS4 glacial isolation, shaped this biogeographic split, with geothermal refugia enabling persistence in high‐altitude wetlands. The contracted IR regions and A/T‐biased SSRs in its chloroplast genome reflect adaptive evolution to dynamic wetland environments, providing key genomic insights for conserving relict species in biodiversity hotspots under climate change.

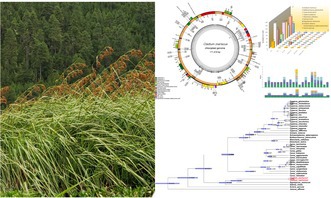

## 1 Introduction

The plants of genus *Cladium* (Cyperaceae) are critical components of wetland ecosystems contributing to oxygen transport and organic matter accumulation, maintaining the stability of this ecological system (Guo et al. 2024; Marazzi et al. 2019; Silvius et al. 2000). Among the *Cladium* species, *C. mariscus* populations exhibit a notably restricted distribution in tropical and subtropical areas. However, recent discoveries have identified outlier populations at high‐altitude and high‐latitude sites in Yunnan and Xizang, China, including Lugu Lake (Ninglang) in the montane area of Yunnan Province and the higher plateau at the entry of Yaluzangbu Valley in Bomi, Xizang, marking the northernmost extent of its known geographic range. These disjunct populations occur within two globally recognized biodiversity hotspots: the Hengduan Mountains and the Yaluzangbu Valley (Duan et al. 2022; Xing and Ree 2017).

The Hengduan Mountains, characterized by their north–south oriented parallel ranges and deeply incised valleys, harbor exceptional biodiversity due to complex topography and climatic gradients (Chang et al. 2025; Ding et al. 2020). Similarly, the Yaluzangbu valley, which originates in the Bomi‐Linzhi region and bisects the Himalayas, hosts a multitude of endemic taxa and serves as a refuge for ancient lineages (Sun et al. 2017). This region exhibits striking biogeographic disjunctions, exemplified by genera such as *Beesia* (Ranunculaceae) and *Dobinea* (Anacardiaceae), which mirror the disjunct pattern observed in *C. mariscus* (Peng et al. 2023). It is still unclear how such disjunct distributions are linked to historical tectonic events, climatic oscillations, or niche conservatisms, offering insights into evolutionary processes shaping biodiversity in topographically complex regions.

Chloroplast (cp) genomes have emerged as pivotal tools for resolving plant phylogenies and reconstructing biogeographic histories due to their structural stability, uniparental inheritance, and low nucleotide substitution rates (Bartczak et al. 2016; Dyall et al. 2004; Yun‐Jie and De‐Zhu 2011). Despite their utility, cp genomes of most Cyperaceae species remain unreachable, especially for species inhabiting ecologically marginal habitats, such as wetlands in higher elevation areas. This study aimed to present the first complete cp genome assembly of a tropical and subtropical wetland species, *C. mariscus* , which was considered to be at the basal position of Cyperaceae, with specimens collected from its disjunct Tibetan and Yunnan populations. By integrating comparative genomics, phylogenetic reconstruction, and molecular dating, we aim to elucidate the evolutionary mechanisms underlying the species' geographic isolation and adaptation to high‐altitude wetlands (Bromham et al. 2018).

## 2 Material and Methods

### 2.1 Sampling and Sequencing

Fresh leaves of *C. mariscus* were collected from two geographically isolated populations (at Lugu Lake, Ninglang (100.84° E, 27.70° N) and Galang Lake, Bomi (95.61° E, 29.91° N)), for which no specific permits are required as it is a common non‐endangered species. The leaves were immediately dried using silica gel to preserve nucleic acid integrity (Chase and Hills 1991). Voucher specimens were archived at the National Wetland Research Center, Southwest Forestry University (accession numbers yue20220061 and yue20220062).

Sequencing libraries were prepared by Personalbio Co. Ltd. (Shanghai, China) using the Illumina TruSeq DNA PCR‐Free Library Preparation Kit. Genomic DNA was fragmented to an average insert size of 350 bp via acoustic shearing (Covaris M220), followed by end repair, adapter ligation, and size selection using AMPure XP beads. Libraries were sequenced on an Illumina NovaSeq 6000 platform with 2 × 150 bp paired‐end reads (Biolabs 2018).

### 2.2 Plastid Genome Assembly and Annotation

Chloroplast genomes were assembled using GetOrganelle v1.7.7 with *H. nemorum* (NC 036036) as the seed reference, employing iterative k‐mer extension to resolve complex plastid architectures. Annotations generated via CPGAVAS2 were manually curated in Geneious Prime 2023.1.2 to validate gene features, intron/exon boundaries, and potential RNA editing sites (Shi et al. 2019). Comparative analyses included: (1) SSR detection across nine Cyperaceae cp genomes using MISA v2.1, and (2) IR boundary characterization through synteny alignment of 39 Cyperaceae plastomes, with junction shifts quantified using IRscope's sliding window approach.

### 2.3 Phylogenomic Reconstruction

A total of 40 chloroplast genomes were used for phylogenetic reconstruction. This dataset included two samples of *C. mariscus* (from Ninglang in Yunnan and Bomi in Tibet), along with 37 species of Cyperaceae and one outgroup ( *Juncus effusus* ), all of which were obtained from NCBI (see Appendix S1). For the structure varied significantly across these genomes, we extracted 98 genes existing among all the genomes and rearranged them into an ideal alignment of orthologous genes using MAFFT automatically with default settings. Phylogenetic trees were reconstructed using the maximum likelihood method implemented in FastTree (Price et al. 2009). These SH values exhibit a significant correlation with conventional bootstrap percentages, reflecting comparable measures of branch reliability in phylogenomic analyses (Shimodaira 2002).

### 2.4 Molecular Dating

Divergence times were estimated using a Bayesian framework implemented in BEAUti and BEAST v2.7.4 (Bouckaert et al. 2014). The maximum likelihood phylogeny, reconstructed from concatenated chloroplast protein‐coding genes, served as the starting tree. Three fossil‐based calibrations were applied to constrain node ages: (1) the root node (Cyperaceae–Juncaceae divergence) was assigned a log‐normal prior with a mean of 60 million years (Ma) and a 95% confidence interval (55–65 Ma), based on early monocot macrofossils (Li et al. 2019); (2) the *Carex* crown node was calibrated to 35.95 Ma (95% HPD: 30–40 Ma) using fossilized *Carex* seeds (Martín‐Bravo et al. 2019); and (3) an internal *Cyperus* node was fixed at 11.8 Ma (95% HPD: 10–13 Ma) derived from paleopalynological evidence (Gao et al. 2025). One independent Markov chain Monte Carlo (MCMC) run of 10 million generations was performed, sampling parameters every 1000 steps. Convergence was assessed in Tracer v1.7.2 (Rambaut et al. 2018), ensuring effective sample sizes for all parameters. The maximum clade credibility tree was generated using TreeAnnotator v2.7.4 after discarding 25% of samples as burn‐in.

## 3 Result

### 3.1 Features of Chloroplast Genome

**FIGURE 1:**
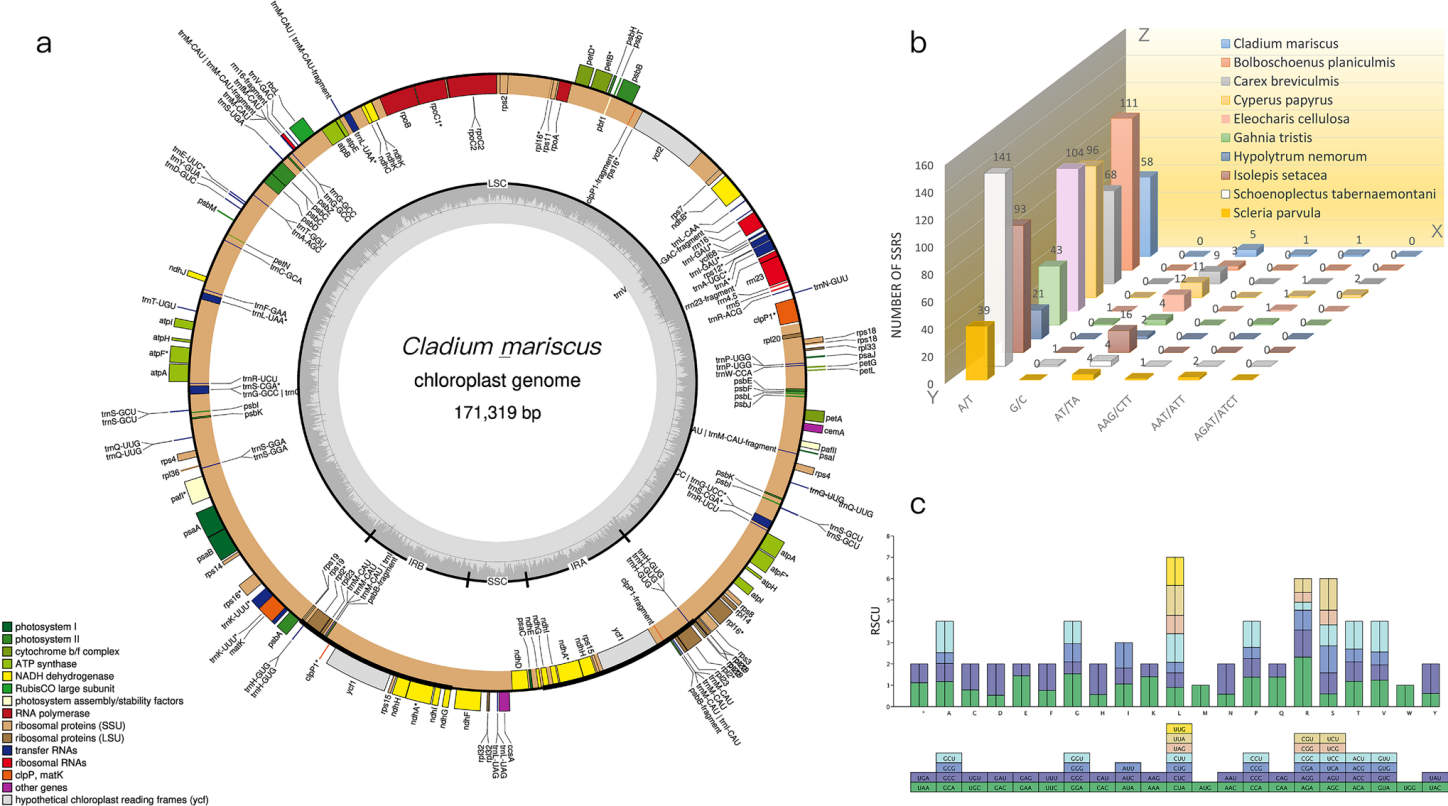
(a) Circular gene map of the *C. mariscus* chloroplast genome. Annotated genes are color‐coded according to their functional categories. Genes outside the circle are transcribed clockwise, while those inside the circle are transcribed counterclockwise. The inner dark gray gradient represents GC content, and the light gray gradient corresponds to AT content. (b) Comparison of the frequency distribution of simple sequence repeats (SSRs) in different species. X‐axis means types of SSRs, Y‐axis means genera of the Cyperaceae, and Z‐axis represents the number of SSRs. (c) Codon usage bias analysis in the *C. mariscus* chloroplast genome. The figure above shows the Relative Synonymous Codon Usage (RSCU) values for the respective amino acids. Colored blocks represent different codons.

The annotated chloroplast genomes of *C. mariscus* from Bomi and Ninglang have been deposited in CNCB (China National Center for Bioinformation) under accession numbers GB0006931 and GB0006905, respectively. For the analysis of its structural features, the complete chloroplast genome of the Bomi specimen spans a total length of 171,319 base pairs (Figure 1) with a GC content of 34.36%. This genome size is consistent with the typical size range observed in most terrestrial plant chloroplast genomes, which generally vary between 120,000 and 170,000 bp (Li et al. 2013). Chloroplast genomes of land plants typically exhibit a quadripartite structure, and *C. mariscus* is no exception. The genome consists of a large single‐copy (LSC) region of 133,558 bp, a small single‐copy (SSC) region of 8059 bp, and two inverted repeat (IR) regions, each 14,851 bp long.

A total of 89 genes encoding 14 proteins were identified in this chloroplast genome (see Appendix S2). The genome further contains a total of 131 genes, which include 89 protein‐coding genes (with 74 being unique), 37 tRNA genes (29 unique), and 5 rRNA genes (4 unique). The presence of protein‐coding genes responsible for photosynthesis, ribosomal, and gene expression reflects the functional conservation of chloroplast genomes across plant species (Sugiura 1992). Moreover, two of these genes, rps12 and clpP, contain two introns, a feature also commonly observed in other chloroplast genomes and associated with regulatory mechanisms that control gene expression at the RNA splicing level (Hu et al. 2015). Introns in chloroplast genes are known to play essential roles in the posttranscriptional regulation of gene expression, further emphasizing the complexity of chloroplast genome evolution (Jacobs and Kück 2011).

### 3.2 SSR and Long Repeats Sequence Analysis

SSRs are widely used as molecular markers due to their high polymorphism, codominant inheritance, and abundance in both coding and noncoding regions of the genome, making them powerful tools for investigating genetic diversity and population structure in plants (Ellegren 2004). The total number of SSRs identified in *C. mariscus* was 65, while in *Bolboschoenus planiculmis* it was 114, *Carex breviculmis* 77, *Cyperus papyrus* 110, *Eleocharis cellulosa* 117, *G. tristis* 49, *Hypolytrum nemorum* 23, *Isolepis setacea* 109, *Schoenoplectus tabernaemontani* 146, and *Scleria parvula* 46 (Figure 1, Appendix S3). The highest SSR count was observed in *S. tabernaemontani* (146), whereas *H. nemorum* exhibited the lowest (23). The A/T mononucleotide repeat was the most abundant motif (774), followed by the AT/TA dinucleotide repeat (70), consistent with findings in other plant species where A/T‐rich motifs dominate SSR composition (Morgante et al. 2002). Among these species, only *Cyperus papyrus* contained tetranucleotide repeats (AGAT/ATCT), a rare occurrence in plant genomes that could be linked to specific evolutionary processes or selective constraints (Kassai‐Jáger et al. 2008). Notably, there was significant variation in the number of A/T mononucleotide repeats across the eight species, ranging from 141 in *S. tabernaemontani* to 21 in *H. nemorum* . The least abundant repeat types were G/C, AAG/CTT, and AGAT/ATCT repeats, each with only two occurrences.

### 3.3 Codon Usage Bias

Codon usage bias, shaped by translational efficiency and accuracy‐related selective pressures, optimizes preferred synonymous codons matching abundant tRNAs in highly expressed genes for rapid translation, while non‐preferred codons contribute to cellular functions like protein folding through mechanisms such as translational pausing (Labella et al. 2019). The usage of the start codon methionine (AUG) and tryptophan (UGG) shows no bias, as indicated by their relative synonymous codon usage (RSCU = 1). Among the preferred synonymous codons (RSCU > 1), the majority end with either adenine (A) or uracil (U), suggesting a tendency toward A/U‐ending codons in this organism. In contrast, within the non‐preferred codons (RSCU < 1), the codon CGC, which codes for phenylalanine, is the least favored. Additionally, no rare codons (RSCU < 0.1) were detected in the genes of *C. mariscus* (Figure 1), indicating a lack of extremely infrequent codon usage in this species.

### 3.4 IR Expansion and Contraction

In chloroplast genomes, Inverted Repeat (IR) regions are generally stable but may undergo expansions (increasing length to drive gene duplication/novel gene emergence) or contractions (reducing length leading to gene loss/sequence simplification), with implications for genomic structural diversity, plant adaptability, and evolutionary trajectories (Hu et al. 2015; Zhu et al. 2016); analysis of species‐specific IR dynamics reveals these structural shifts significantly influence gene arrangement, expression, and adaptive functions in chloroplast genome evolution (Wang et al. 2021; Zhang et al. 2024).

**FIGURE 2:**
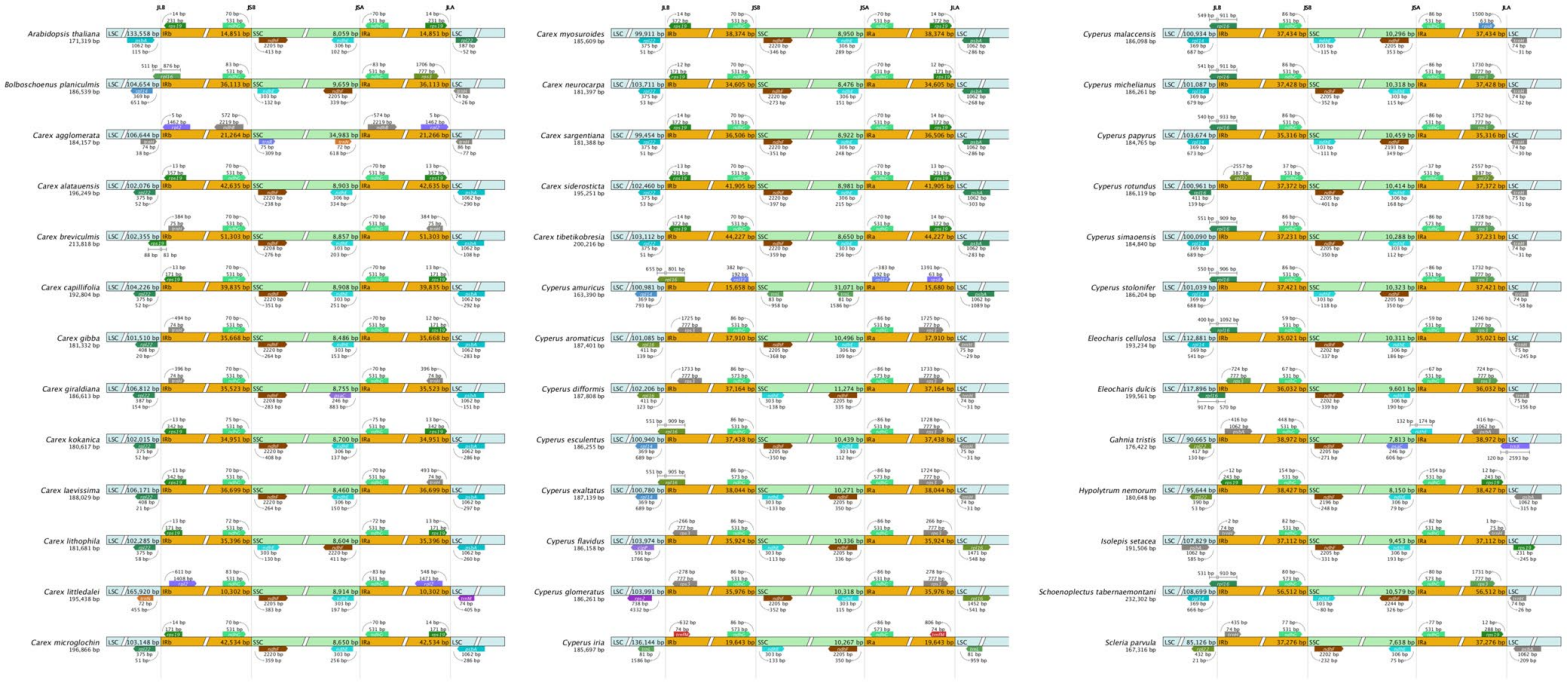
Comparison of the border positions of LSC, SSC, and IR regions among the 45 chloroplast genomes of Cyperaceae species. Genes are represented by boxes, with the gene name and transcriptional extent indicated above. The gap between a gene and the boundary, unless the gene coincides with the boundary, is indicated by the number of base pairs. JLB: LSC/IRb; JSB: IRb/SSC; JSA: SSC/IRa; JLA: IRa/LSC.

The results indicate that *C. mariscus* exhibits a notable contraction of the IR regions (IRA and IRB) compared to other Cyperaceae species, with a length of only 29,702 bp, while the LSC (Large Single Copy) region is significantly longer than in other species of the same family (Figure 2). There are marked differences in the boundary regions among other Cyperaceae species, with several showing considerable IR expansion, such as in the genus *Carex*, particularly in *C. breviculmis*. The IR region of *C. breviculmis* spans a total of 102,606 bp, with the rps19 gene crossing the LSC/IRb boundary and extending 88 bp into the LSC. In contrast, the genus Cyperus demonstrates a contraction relative to *Carex*, which is evident in the rpl16 gene at the LSC/IRb junction, where the contraction of rpl16 ranges between 905 and 933 bp.

### 3.5 Phylogenomic Reconstruction and Divergence Time Dating

**FIGURE 3:**
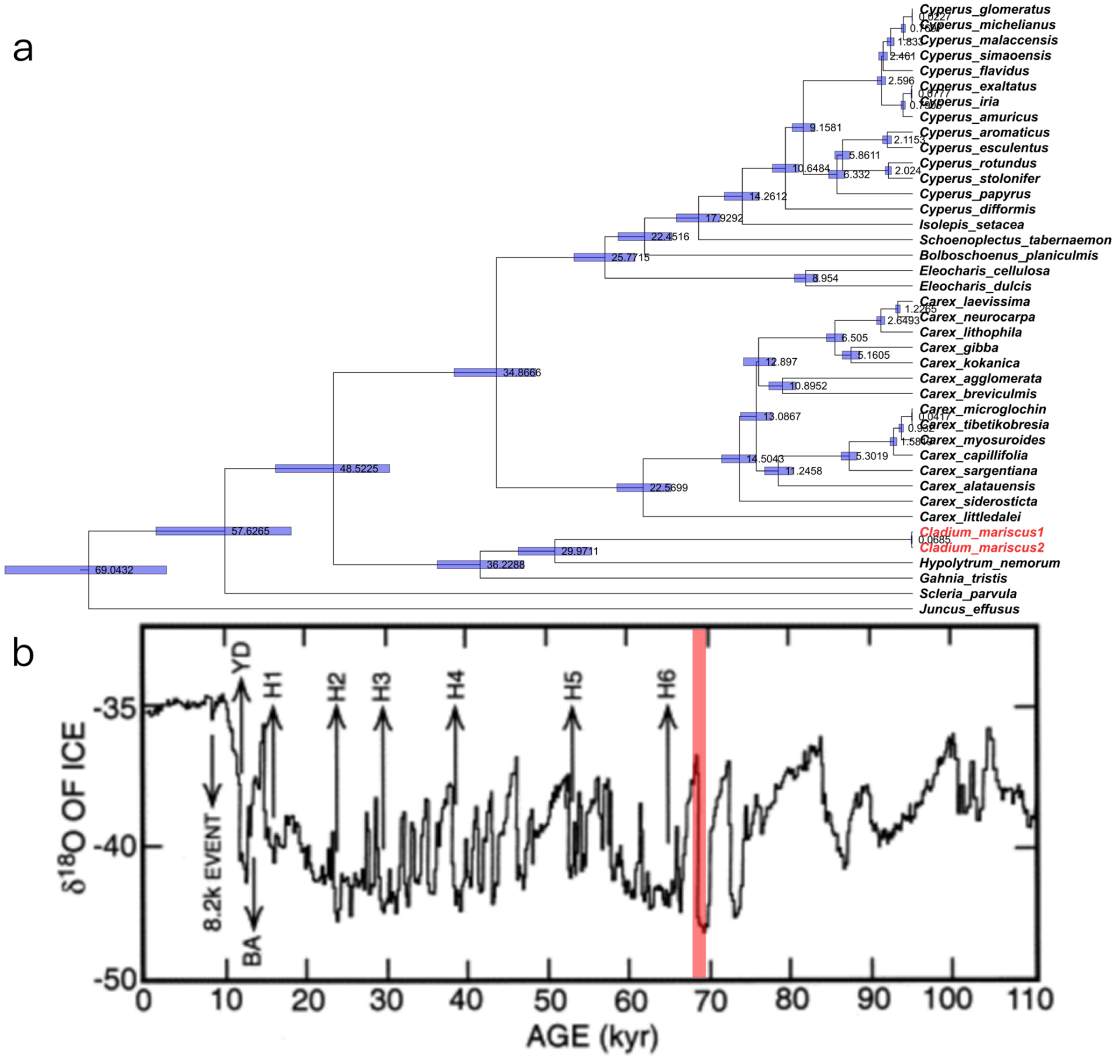
(a) A relaxed molecular clock analysis estimating the divergence times within the study group. Tips in red font denote the two *C. mariscus* lineages, which diverged at 68.5 thousand years ago (kya). The blue bars represent the 95% confidence intervals of diverging time scales among. (b) Northern Hemisphere paleotemperature variations around 68 ka, based on the GRIP ice core oxygen isotope record (δ^18^O) from Greenland (Bond et al. 1993; Broecker 2000). The period around 68 ka corresponds to a cold stadial phase within Dansgaard‐Oeschger cycle 18, characterized by a general cooling trend and increased climate instability preceding a potential abrupt warming event. The region highlighted in red corresponds to approximately 68 ka, during which a sharp decline in δ^18^O values is observed, indicating a significant decrease in temperature.

Cyperaceae exhibits substantial genomic divergence, likely attributable to its adaptive radiation and evolutionary plasticity (Escudero and Hipp 2013). There are a large number of losses and variations in the Cyperaceae genes (Table 1). Phylogenetic relationships within the Cyperaceae were reconstructed using the Maximum Likelihood (ML) method, which resolved *H. nemorum* and *Gahnia tristis* as sister species to *C. mariscus* (Figure 3). To distinguish between the two geographically distinct samples, we refer to the specimen from Galang Lake, Bomi as *C. mariscus*1 and the specimen from Lugu Lake, Ninglang as *C. mariscus*2 hereafter.

**TABLE 1:** There are significant differences and missing genes among the 40 species.

	Missing gene	Genes with significant differences
ndhF	*Cyperus amuricus*	
psbN		*Carex littledalei*, *C. amuricus* , *Cyperus simaoensis*
rpoC2		*S. tabernaemontani*
ycf3		*C. mariscus1* , *C*. *breviculmis* *Carex lithophila*, *Carex tibetikobresia*
trnG‐UCC	*C. lithophila*	
trnfM‐CAU	*Cyperus aromaticu*s, *Cyperus rotundus*	

Molecular dating inferred Cyperaceae‐Juncaceae divergence at ~69 Ma, with *Carex* crown diversification initiating at 34.87 Ma and *Cyperus* radiation commencing at 14.26 Ma. Critically, the two *C. mariscus* lineages from Ninglang and Bomi diverged approximately 68.5 thousand years ago (ka), coinciding with the MIS4 of late Pleistocene climatic oscillations (Figure 3). This divergence event occurred during a period of profound Northern Hemisphere cooling and climatic instability, as recorded in Greenland ice cores (e.g., GRIP), which showed markedly depressed δ^18^O values indicative of severe stadial conditions (Johnsen et al. 1995). The concurrent timing suggests a potential link between population fragmentation in *C. mariscus* and the extreme glacial climate characterizing this interval.

## 4 Discussion

### 4.1 Feature of *Cladium mariscus* 's Chloroplast Genome

While possessing the typical tetrad chloroplast structure common to angiosperms (Jensen and Leister 2014), *C. mariscus* exhibits a significantly contracted inverted repeat (IRA & IRB) region (29,702 bp), in contrast to the expanded IRA & IRB in C. breviculmis (102,606 bp) (Hereafter, ‘IR’ refers to the combined IRA and IRB regions.). This divergent IR architecture suggests distinct evolutionary trajectories within the family, potentially influencing genome stability and gene retention. Specifically, the IR contraction in *C. mariscus* may reflect ecological niche specialization in dynamic wetland habitats, differing from genomic adaptations potentially linked to reproductive strategies in *Carex* (Guo et al. 2023).

In codon usage bias and simple sequence repeat (SSR) composition, *C. mariscus* aligns with broader monocot patterns but shows potential lineage‐specific optimization. The dominance of A/T mononucleotide SSRs (89.2%) is consistent with findings in other monocots (Lawson and Zhang 2006; Wang et al. 2018; Zhou et al. 2016). Similarly, the absence of rare codons (RSCU ≥ 0.1) and strong preference for A/U‐ending codons likely indicate selective pressures for translational efficiency—a trend observed across high‐expression photosynthetic genes in plants (Gingold and Pilpel 2011). However, the degree of bias in *C. mariscus* may reflect specific adaptations to its metabolic demands in wetland environments.

Phylogenetically, *C. mariscus* displays unexpected relationships and conservation despite ecological divergence. The species forms a monophyletic clade sister to *Hypolytrum nemorum*, contradicting prior morphology‐based taxonomy and suggesting potential convergent floral evolution or incomplete lineage sorting. Notably, minimal cpDNA divergence exists between geographically isolated populations (Bomi vs. Ninglang), despite occupying distinct high‐altitude Tibetan and tropical Yunnanese habitats. This contrasts with the marked cp genome divergence observed in *C*. *breviculmis* (Jiménez‐Mejías et al. 2016), implying either recent dispersal in *C. mariscus* or strong stabilizing selection on its chloroplast genome.

### 4.2 Latest Divergence Between the Two Lineages

Molecular dating indicates a divergence time of approximately 70 ka for the Bomi and Hengduan lineages, indicating a vicariance evolution pattern by montane landscape, coinciding with a cooling down period during the MIS4. The exceptionally warm glacial intervals enabled *C. mariscus* 's northward expansion across a contiguous network of Himalayan wetlands, facilitated by intensified monsoonal precipitation and enhanced meltwater inputs from glacial sources (Zhou et al. 2006). Throughout this extended thermal optimum (~130–70 ka), the species likely maintained a pan‐regional distribution characterized by substantial gene flow. This pattern is also reported as shared ancestral chloroplast haplotypes across populations and coalescent simulations indicating an effective population size 5–7 times larger than that observed in modern relict populations prior to the Last Glacial Maximum (Doughty et al. 2021).

Connectivity collapsed during late Pleistocene cooling (~70–15 ka), as temperature declines of 5°C–7°C compressed thermal niches, forcing populations into fragmented valley refugia (Chen et al. 2008; Selwood and Zimmer 2020). This fragmentation was preconditioned by tectonically controlled landscapes: fault‐driven incision of the Jinshajiang and Nujiang valleys severed critical wetland corridors, while geothermal anomalies within rift zones (notably the Bomi graben) buffered microclimates, allowing tropical‐affiliated lineages to persist at temperatures 3°C–5°C above regional averages (Sun et al. 2017). Although pluvial phases during MIS 3 (~57–29 ka) briefly reconnected habitats through expansions of paleolake systems (Li et al. 2008), the topographic barrier of the Hengduan Mountains ultimately enforced profound genetic divergence between isolated populations.

Lineage‐specific mutations accumulated after ~18 ka reflect a lagged genomic response to prolonged glacial isolation, coinciding with population stabilization in relict wetlands. This integrative narrative reconciles the timing of genetic divergence with paleoenvironmental archives, emphasizing how pre‐LGM (MIS5–3) climatic warmth seeded broad species distributions, while tectonic topography and late Pleistocene climatic extremes sculpted modern biogeographic disjunctions. The resulting framework is applicable to other monsoon‐dependent relict species across Asian biodiversity hotspots.

## 5 Conclusions

This study resolves the phylogeographic paradox of *C. mariscus* 's disjunct alpine populations through integrated plastid genomics and molecular dating. Our findings demonstrate a Late Pleistocene lineage divergence (~68.5 ka) between Tibetan (Bomi) and Hengduan (Ninglang) populations, temporally decoupled from the Miocene–Pliocene tectonic fragmentation of these biodiversity hotspots. Instead, Quaternary glacial–interglacial cycles drove genetic isolation via cyclical habitat fragmentation. The species' persistence in high‐altitude refugia reflects niche conservatism and geothermal microclimate buffering, enabling tropical‐affiliated lineages to colonize temperate alpine wetlands. Notably, chloroplast genomic features including contracted IR regions and A/T‐biased SSRs underscore evolutionary adaptations to dynamic wetland ecosystems. These results recalibrate the relative roles of climate oscillations versus tectonic processes in shaping biodiversity hotspot disjunctions, while providing critical genomic baselines for conserving relic wetland taxa under accelerating climate change. Future studies should explore nuclear‐genomic discordance and functional implications of plastid structural variation in Cyperaceae adaptive radiation.

## Author Contributions

**Xin‐Hui Qian:** data curation (lead), formal analysis (lead), investigation (lead), methodology (lead), writing – original draft (lead). **Liang‐Liang Yue:** conceptualization (lead), funding acquisition (lead), supervision (lead), writing – review and editing (lead).

## Funding

This work was supported by the Applied Basic Research Key Project of Yunnan Province, 202301AS070001, National Natural Science Foundation of China, 42161015.

## Conflicts of Interest

The authors declare no conflicts of interest.

## Supporting information


**Appendix S1:** 38 complete chloroplast sequence species obtained on NCBI.

**Appendix S2:** The 89 genetic classifications of *Cladium mariscus*.

**Appendix S3:** The detailed number and total number of simple sequence repeats (SSRs) of different species.

## Data Availability

All the required data is uploaded as [Link], [Link], [Link].

## References

[ece372456-bib-0001] Bartczak, P. , S. Żółtowska , M. Norman , et al. 2016. “Saw‐Sedge *Cladium mariscus* as a Functional Low‐Cost Adsorbent for Effective Removal of 2, 4‐Dichlorophenoxyacetic Acid From Aqueous Systems.” Adsorption 22: 517–529.

[ece372456-bib-0002] Biolabs, N. E. 2018. “Ultra II DNA Library Prep Kit for Illumina E7645/E7103.”

[ece372456-bib-0003] Bond, G. , W. Broecker , S. Johnsen , et al. 1993. “Correlations Between Climate Records From North Atlantic Sediments and Greenland Ice.” Nature 365, no. 6442: 143–147.

[ece372456-bib-0004] Bouckaert, R. , J. Heled , D. Kühnert , et al. 2014. “BEAST 2: A Software Platform for Bayesian Evolutionary Analysis.” PLoS Computational Biology 10, no. 4: e1003537. 24722319 10.1371/journal.pcbi.1003537 PMC3985171

[ece372456-bib-0005] Broecker, W. 2000. “Abrupt Climate Change: Causal Constraints Provided by the Paleoclimate Record.” Earth‐Science Reviews 51, no. 1–4: 137–154.

[ece372456-bib-0006] Bromham, L. , S. Duchêne , X. Hua , A. M. Ritchie , D. A. Duchêne , and S. Y. Ho . 2018. “Bayesian Molecular Dating: Opening Up the Black Box.” Biological Reviews 93, no. 2: 1165–1191. 29243391 10.1111/brv.12390

[ece372456-bib-0007] Chang, Y. , K. Gelwick , X. Fang , et al. 2025. “Tectonic and Climatic Controls on Plant Biodiversity in the Hengduan Mountains, China.” Geological Society, London, Special Publications 549, no. 1: 205–225. 10.1144/sp549-2024-17.

[ece372456-bib-0008] Chase, M. W. , and H. H. Hills . 1991. “Silica Gel: An Ideal Material for Field Preservation of Leaf Samples for DNA Studies.” Taxon 40, no. 2: 215–220.

[ece372456-bib-0009] Chen, S. , G. Wu , D. Zhang , et al. 2008. “Potential Refugium on the Qinghai–Tibet Plateau Revealed by the Chloroplast DNA Phylogeography of the Alpine Species Metagentiana Striata (Gentianaceae).” Botanical Journal of the Linnean Society 157, no. 1: 125–140.

[ece372456-bib-0010] Ding, W.‐N. , R. H. Ree , R. A. Spicer , and Y.‐W. Xing . 2020. “Ancient Orogenic and Monsoon‐Driven Assembly of the World's Richest Temperate Alpine Flora.” Science 369, no. 6503: 578–581. 32732426 10.1126/science.abb4484

[ece372456-bib-0011] Doughty, A. M. , M. R. Kaplan , C. Peltier , and S. Barker . 2021. “A Maximum in Global Glacier Extent During MIS 4.” Quaternary Science Reviews 261: 106948.

[ece372456-bib-0012] Duan, S. , F. Han , F. Li , and Z. Yang . 2022. “Spatial Evaluation of the Ecological Value Importance of National Park in Yarlung Tsangpo Grand Canyon.” Journal of Environmental Management 320: 115943. 10.1016/j.jenvman.2022.115943. 36056501

[ece372456-bib-0013] Dyall, S. D. , M. T. Brown , and P. J. Johnson . 2004. “Ancient Invasions: From Endosymbionts to Organelles.” Science 304, no. 5668: 253–257. 15073369 10.1126/science.1094884

[ece372456-bib-0014] Ellegren, H. 2004. “Microsatellites: Simple Sequences With Complex Evolution.” Nature Reviews Genetics 5, no. 6: 435–445. 10.1038/nrg1348 15153996

[ece372456-bib-0015] Escudero, M. , and A. Hipp . 2013. “Shifts in Diversification Rates and Clade Ages Explain Species Richness in Higher‐Level Sedge Taxa (Cyperaceae).” American Journal of Botany 100, no. 12: 2403–2411. 24249788 10.3732/ajb.1300162

[ece372456-bib-0016] Gao, Z. , Y. Cai , J. Long , B. Wang , Z. Huang , and Y. Gao . 2025. “The Complete Chloroplast Genome and the Phylogenetic Analysis of *Fimbristylis littoralis* (Cyperaceae) Collected in Cherry Blossom Nursery.” International Journal of Molecular Sciences 26, no. 5: 2321. 40076940 10.3390/ijms26052321 PMC11901024

[ece372456-bib-0017] Gingold, H. , and Y. Pilpel . 2011. “Determinants of Translation Efficiency and Accuracy.” Molecular Systems Biology 7, no. 1: 481. 21487400 10.1038/msb.2011.14 PMC3101949

[ece372456-bib-0018] Guo, M. , W.‐L. Xue , C. Wang , et al. 2024. “Environmental Flow Increases the Riparian Vegetation Diversity and Community Similarity.” Wetlands 44, no. 5: 57. 10.1007/s13157-024-01811-w.

[ece372456-bib-0019] Guo, X. , F. Wang , D. Fang , et al. 2023. “The Genome of Acorus Deciphers Insights Into Early Monocot Evolution.” Nature Communications 14, no. 1: 3662. 10.1038/s41467-023-38836-4 PMC10281966 37339966

[ece372456-bib-0020] Hu, S. , G. Sablok , B. Wang , et al. 2015. “Plastome Organization and Evolution of Chloroplast Genes in *Cardamine* Species Adapted to Contrasting Habitats.” BMC Genomics 16: 1–14. 25887666 10.1186/s12864-015-1498-0 PMC4446112

[ece372456-bib-0021] Jacobs, J. , and U. Kück . 2011. “Function of Chloroplast RNA‐Binding Proteins.” Cellular and Molecular Life Sciences 68: 735–748. 20848156 10.1007/s00018-010-0523-3 PMC11115000

[ece372456-bib-0022] Jensen, P. E. , and D. Leister . 2014. “Chloroplast Evolution, Structure and Functions.” F1000Prime Reports 6: 40. 24991417 10.12703/P6-40 PMC4075315

[ece372456-bib-0023] Jiménez‐Mejías, P. , M. Hahn , K. Lueders , et al. 2016. “Megaphylogenetic Specimen‐Level Approaches to the Carex (Cyperaceae) Phylogeny Using ITS, ETS, and matK Sequences: Implications for Classification.” Systematic Botany 41, no. 3: 500–518.

[ece372456-bib-0024] Johnsen, S. J. , D. Dahl‐Jensen , W. Dansgaard , and N. Gundestrup . 1995. “Greenland Palaeotemperatures Derived From GRIP Bore Hole Temperature and Ice Core Isotope Profiles.” Tellus B: Chemical and Physical Meteorology 47, no. 5: 624–629.

[ece372456-bib-0025] Kassai‐Jáger, E. , C. Ortutay , G. Tóth , T. Vellai , and Z. Gáspári . 2008. “Distribution and Evolution of Short Tandem Repeats in Closely Related Bacterial Genomes.” Gene 410, no. 1: 18–25. 18191346 10.1016/j.gene.2007.11.006

[ece372456-bib-0026] Labella, A. L. , D. A. Opulente , J. L. Steenwyk , C. T. Hittinger , and A. Rokas . 2019. “Variation and Selection on Codon Usage Bias Across an Entire Subphylum.” PLoS Genetics 15, no. 7: e1008304. 31365533 10.1371/journal.pgen.1008304 PMC6701816

[ece372456-bib-0027] Lawson, M. J. , and L. Zhang . 2006. “Distinct Patterns of SSR Distribution in the *Arabidopsis thaliana* and Rice Genomes.” Genome Biology 7: 1–11. 10.1186/gb-2006-7-2-r14 PMC1431726 16507170

[ece372456-bib-0028] Li, H.‐T. , T.‐S. Yi , L.‐M. Gao , et al. 2019. “Origin of Angiosperms and the Puzzle of the Jurassic Gap.” Nature Plants 5, no. 5: 461–470. 31061536 10.1038/s41477-019-0421-0

[ece372456-bib-0029] Li, S. , H. Zhang , Y. Shi , and Z. Zhu . 2008. “A High Resolution MIS 3 Environmental Change Record Derived From Lacustrine Deposit of Tianshuihai Lake, Qinghai‐Tibet Plateau.” Quaternary Sciences 28, no. 1: 122–131.

[ece372456-bib-0030] Li, X. , H. Gao , Y. Wang , et al. 2013. “Complete Chloroplast Genome Sequence of Magnolia Grandiflora and Comparative Analysis With Related Species.” Science China Life Sciences 56: 189–198. 23329156 10.1007/s11427-012-4430-8

[ece372456-bib-0031] Marazzi, L. , E. E. Gaiser , M. B. Eppinga , et al. 2019. “Why Do We Need to Document and Conserve Foundation Species in Freshwater Wetlands?” Water 11, no. 2: 265.

[ece372456-bib-0032] Martín‐Bravo, S. , P. Jiménez‐Mejías , T. Villaverde , et al. 2019. “A Tale of Worldwide Success: Behind the Scenes of *Carex* (Cyperaceae) Biogeography and Diversification.” Journal of Systematics and Evolution 57, no. 6: 695–718.

[ece372456-bib-0033] Morgante, M. , M. Hanafey , and W. Powell . 2002. “Microsatellites Are Preferentially Associated With Nonrepetitive DNA in Plant Genomes.” Nature Genetics 30, no. 2: 194–200. 11799393 10.1038/ng822

[ece372456-bib-0035] Peng, J. , X. Ma , and H. Sun . 2023. “Ancient Allopatry and Ecological Divergence Act Together to Promote Plant Diversity in Mountainous Regions: Evidence From Comparative Phylogeography of Two Genera in the Sino‐Himalayan Region.” BMC Plant Biology 23, no. 1: 572. 37978437 10.1186/s12870-023-04593-1 PMC10655281

[ece372456-bib-0036] Price, M. N. , P. S. Dehal , and A. P. Arkin . 2009. “FastTree: Computing Large Minimum Evolution Trees With Profiles Instead of a Distance Matrix.” Molecular Biology and Evolution 26, no. 7: 1641–1650. 19377059 10.1093/molbev/msp077 PMC2693737

[ece372456-bib-0037] Rambaut, A. , A. J. Drummond , D. Xie , G. Baele , and M. A. Suchard . 2018. “Posterior Summarization in Bayesian Phylogenetics Using Tracer 1.7.” Systematic Biology 67, no. 5: 901–904. 29718447 10.1093/sysbio/syy032 PMC6101584

[ece372456-bib-0038] Selwood, K. , and H. Zimmer . 2020. “Refuges for Biodiversity Conservation: A Review of the Evidence.” Biological Conservation 245: 108502.

[ece372456-bib-0039] Shi, L. , H. Chen , M. Jiang , et al. 2019. “CPGAVAS2, an Integrated Plastome Sequence Annotator and Analyzer.” Nucleic Acids Research 47, no. W1: W65–W73. 31066451 10.1093/nar/gkz345 PMC6602467

[ece372456-bib-0040] Shimodaira, H. 2002. “An Approximately Unbiased Test of Phylogenetic Tree Selection.” Systematic Biology 51, no. 3: 492–508. 12079646 10.1080/10635150290069913

[ece372456-bib-0041] Silvius, M. J. , M. Oneka , and A. Verhagen . 2000. “Wetlands: Lifeline for People at the Edge.” Physics and Chemistry of the Earth, Part B: Hydrology, Oceans and Atmosphere 25, no. 7: 645–652. 10.1016/S1464-1909(00)00079-4.

[ece372456-bib-0042] Sugiura, M. 1992. “The Chloroplast Genome.” Plant Molecular Biology 19: 149–168. 1600166 10.1007/BF00015612

[ece372456-bib-0043] Sun, H. , J. Zhang , T. Deng , and D. E. Boufford . 2017. “Origins and Evolution of Plant Diversity in the Hengduan Mountains, China.” Plant Diversity 39: 161–166. 30159507 10.1016/j.pld.2017.09.004 PMC6112316

[ece372456-bib-0044] Wang, H. , Y. Lei , L. Yan , et al. 2018. “Development and Validation of Simple Sequence Repeat Markers From *Arachis hypogaea* Transcript Sequences.” Crop Journal 6, no. 2: 172–180.

[ece372456-bib-0045] Wang, M. , X. Wang , J. Sun , et al. 2021. “Phylogenomic and Evolutionary Dynamics of Inverted Repeats Across Angelica Plastomes.” BMC Plant Biology 21: 1–12. 33413122 10.1186/s12870-020-02801-w PMC7792290

[ece372456-bib-0046] Xing, Y. , and R. H. Ree . 2017. “Uplift‐Driven Diversification in the Hengduan Mountains, a Temperate Biodiversity Hotspot.” Proceedings of the National Academy of Sciences of the United States of America 114, no. 17: E3444–E3451. 10.1073/pnas.1616063114. 28373546 PMC5410793

[ece372456-bib-0047] Yun‐Jie, Z. , and L. De‐Zhu . 2011. “Advances in Phylogenomics Based on Complete Chloroplast Genomes.” Plant Diversity 33, no. 4: 365–375.

[ece372456-bib-0048] Zhang, Y. , M. Song , D. Tang , et al. 2024. “Comprehensive Comparative Analysis and Development of Molecular Markers for Lasianthus Species Based on Complete Chloroplast Genome Sequences.” BMC Plant Biology 24, no. 1: 867. 39285331 10.1186/s12870-024-05383-z PMC11406864

[ece372456-bib-0049] Zhou, S. , X. Wang , J. Wang , and L. Xu . 2006. “A Preliminary Study on Timing of the Oldest Pleistocene Glaciation in Qinghai–Tibetan Plateau.” Quaternary International 154: 44–51.

[ece372456-bib-0050] Zhou, X. , Y. Dong , J. Zhao , et al. 2016. “Genomic Survey Sequencing for Development and Validation of Single‐Locus SSR Markers in Peanut (*Arachis hypogaea* L.).” BMC Genomics 17: 1–14. 27251557 10.1186/s12864-016-2743-x PMC4888616

[ece372456-bib-0051] Zhu, A. , W. Guo , S. Gupta , W. Fan , and J. P. Mower . 2016. “Evolutionary Dynamics of the Plastid Inverted Repeat: The Effects of Expansion, Contraction, and Loss on Substitution Rates.” New Phytologist 209, no. 4: 1747–1756. 26574731 10.1111/nph.13743

